# Evaluation of Single Column Trapping/Separation and Chemiluminescence Detection for Measurement of Methanethiol and Dimethyl Sulfide from Pig Production

**DOI:** 10.1155/2012/489239

**Published:** 2012-09-10

**Authors:** Michael Jørgen Hansen, Kei Toda, Tomoaki Obata, Anders Peter S. Adamsen, Anders Feilberg

**Affiliations:** ^1^Department of Engineering, Science and Technology, Aarhus University, Blichers Allé 20, 8830 Tjele, Denmark; ^2^Department of Chemistry, Kumamoto University, 2-39-1 Kurokami, Kumamoto 860-8555, Japan

## Abstract

Reduced sulfur compounds are considered to be important odorants from pig production due to their low odor threshold values and low solubility in slurry. The objective of the present study was to investigate the use of a portable method with a single silica gel column for trapping/separation coupled with chemiluminescence detection (SCTS-CL) for measurement of methanethiol and dimethyl sulfide in sample air from pig production. Proton-transfer-reaction mass spectrometry (PTR-MS) was used to evaluate the trapping/separation. The silica gel column used for the SCTS-CL efficiently collected hydrogen sulfide, methanethiol and dimethyl sulfide. The measurement of methanethiol by SCTS-CL was clearly interfered by the high concentration of hydrogen sulfide found in pig production, and a removal of hydrogen sulfide was necessary to obtain reliable results. Air samples taken from a facility with growing-finishing pigs were analyzed by SCTS-CL, PTR-MS, and a gas chromatograph with sulfur chemiluminescence detection (GC-SCD) to evaluate the SCTS-CL. The difference between the concentrations of methanethiol and dimethyl sulfide measured with SCTS-CL, PTR-MS, and GC-SCD was below 10%. In conclusion, the SCTS-CL is a portable and low-cost alternative to the commercial methods that can be used to measure methanethiol and dimethyl sulfide in sample air from pig production.

## 1. Introduction

Odor from modern intensive pig production can cause serious nuisance to people living in the vicinity of the facilities. Volatile reduced sulfur compounds are considered to have a significant influence on odor from pig production due to their low odor threshold values [1] and low solubility in slurry. The most abundant sulfur compounds found in pig production are hydrogen sulfide, methanethiol, and dimethyl sulfide [2, 3]. The concentration of hydrogen sulfide in pig production is normally several hundred ppb_v_, whereas the concentration of methanethiol and dimethyl sulfide is in the low ppb_*v*_ range (1–20 ppb_*v*_). Based on the reported odor threshold values [1], particularly hydrogen sulfide and methanethiol may have a large influence on odor from pig production.

Different methods have been applied for analyzing sulfur compounds in sample air from animal production. Online measurements by proton-transfer-reaction mass spectrometry (PTR-MS) have been used to measure sulfur compounds and other odorants from pig production [2, 3] and cattle production [4, 5]. Gas chromatography combined with a sulfur-specific detector such as flame photometric detection (GC-FPD), pulsed flame photometric detection (GC-PFPD), and sulfur chemiluminescence detection (GC-SCD) is often used to measure sulfur compounds in air. Pandey and Kim [6] reviewed these GC methods for the reduced sulfur compounds. Measurement of sulfur compounds from pig production with sulfur-specific detectors requires the use of sampling bags or preconcentration of sample air on adsorbent tubes or adsorptive fibers. Sulfur compounds can be stored in Tedlar bags for several hours with a recovery above 90–95% [7–10], but this sampling method requires a detector with a detection limit in the low ppb_*v*_ range or a preconcentration step prior to analysis. Pre-concentration of sample air on adsorbent tubes or adsorptive fibers at the source can avoid the potential loss of sulfur compounds in sample bags, however, graphitized and carbon molecular sieve sorbents and to some extent Tenax TA result in oxidation of thiols into disulfides [11, 12]. Compared to graphitized and carbon molecular sieve sorbents and Tenax TA, the use of silica gel for pre-concentration of sulfur compounds has been shown to have a higher recovery of hydrogen sulfide (78.7%) [13] and methanethiol (98.2%) [14], but it requires drying of the sample air to avoid water sorption. The advantage of silica gel for pre-concentration of sulfur compounds was used to develop a method with a single column for trapping/separation and chemiluminescence detection (SCTS-CL) [15]. Sulfur chemiluminescence detection normally includes a conversion of sulfur compounds into SO in a hydrogen/air burner, reaction with ozone, and detection of the chemiluminescent emission from excited SO_2_*. However, even without the conversion step, reduced sulfur compounds react with ozone, thus resulting in a chemiluminescent emission that can be detected [16, 17]. This type of chemiluminescence detection was applied in the SCTS-CL, where separated sulfur compounds from the silica gel column and ozone were mixed, and the chemiluminescent emission was detected by a photomultiplier. Although other measurement techniques are available for measurement of sulfur compounds, the SCTS-CL is a portable and low-cost (ca. 5,000 $) method that could be an alternative to the commercial methods. The SCTS-CL has previously been applied to atmospheric analysis of sulfur gases [18] and isoprene [19] and analysis of isoprene contained in breath [20], but the method has not been applied to ventilation air from pig production or other types of animal production. Ventilation air from animal production is generally characterized by high levels of dust, moisture, and hydrogen sulfide, and the performance of SCTS-CL should therefore be tested under these specific conditions.

The objective of the present study was (i) to optimize the SCTS-CL for measuring methanethiol and dimethyl sulfide under the conditions present in sample air from pig production and (ii) to perform an intercomparison between SCTS-CL, PTR-MS, and GC-SCD with sample air from a pig production facility with growing-finishing pigs.

## 2. Materials and Methods

### 2.1. Chemicals and Materials

Silica gel used for gas collection and separation was Davison grade 12, 60/80 mesh (Supelco, Bellefonte, PA, USA). The silica gel was packed in a ceramic tube (4 mm i.d. × 6 mm o.d. × 100 mm). The effective adsorbent bed was 6 cm long, and the ends were plugged with silanized quartz wool. A nickel-chrome wire (0.2 mm × 35 cm, Nilaco, Tokyo, Japan) was coiled around the ceramic tube and used to heat the column. The nickel-chrome wire was covered by glass wool and aluminum tape.

The linearity of the SCTS-CL was estimated based on gas mixture 1 (Table 1). Desorption of compounds, trapping efficiency, and the interferences between sulfur compounds for the SCTS-CL were investigated by PTR-MS. Gas mixture 2 and 3 (Table 1) were used to investigate the desorption of methanethiol and dimethyl sulfide, and gas mixture 4 (Table 1) was used to investigate the trapping efficiency and the interferences between compounds.

### 2.2. SCTS-CL

The SCTS-CL for the sulfur gases was developed at Kumamoto University, Japan [15] and was improved by Toda et al. [18]. The SCTS-CL was constructed at Kumamoto University by the lead author and was subsequently applied in Denmark. The instrument was composed of two parts which were (i) a silica gel column used for trapping and separation of sulfur compounds and (ii) a chemiluminescence detector. The chemiluminescence cell was made of a stainless steel tube (32 mm i.d. × 40 mm) where one end was capped with a stainless steel plate with an inlet/outlet tube, and the other side was covered with an o-ring sealed glass plate for optical window. Sample air from the column and ozone was introduced to the reaction cell through stainless steel tubes. An ozone generator (1000BT-12 (300 mg h^−1^), Shanghai ENALY M&E Ltd., Shanghai, China) with a flow of 150 mL min^−1^ charcoal filtered atmospheric air was used to generate ozone. A photomultiplier (R3550A, Hamamatsu Photonics, Hamamatsu, Japan) was used to detect the chemiluminescent emission from the reaction cell.

**Figure 1:**
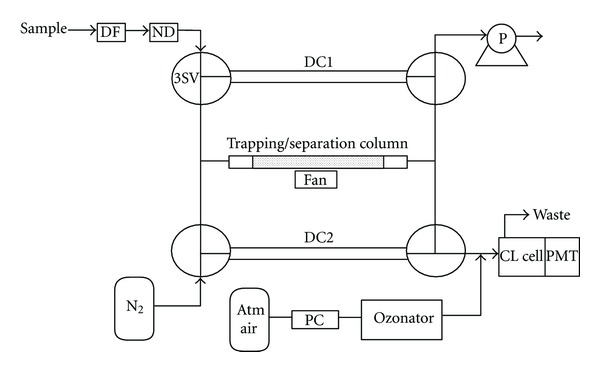
Schematic drawing of single column trapping/separation and chemiluminescence detection (SCTS-CL). DF: dust filter; ND: Nafion dryer; 3SV: 3-way solenoid valve; DC1,2: dummy columns; Atm air: atmospheric air; PC: purification column packed with charcoal; CL cell: chemiluminescence cell; PMT: Photomultiplier; P: air pump.

**Figure 2:**
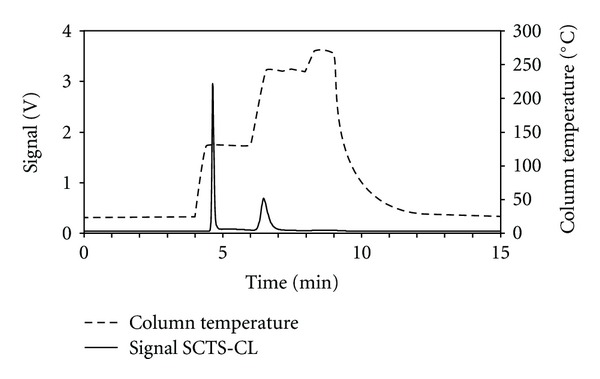
Measurement cycle for single column trapping/separation and chemiluminescence detection (SCTS-CL). Step 1, 0–3 min: sample collection; step 2, 3-4 min: purging (ambient temperature), 4–6 min: measurement of methanethiol (130°C), 6–8 min: measurement of dimethyl sulfide (240°C), 8-9 min: cleaning of the column (270°C); step 3, 9–15 min: cooling of the column. Signals were obtained for ca. 11 ppb_*v*_ methanethiol and dimethyl sulfide.

The silica gel column and the chemiluminescence detector were integrated in a system with four three-way solenoid valves (VT307Y-6G-01, SMC, Tokyo, Japan) that were used to change between the trapping and the separation mode, see Figure 1. Each measurement cycle took 15 min and was divided into three steps, see Figure 2. In step 1, sample air was collected on the column for 3 min with a sample flow at 200 mL min^−1^. The sample air was passed through a 5 *μ*m Teflon particle filter (Millipore, Billerica, MA, USA) and a Nafion dryer (MD-110-48P-4, Perma Pure, Toms River, NJ, USA) prior to the collection on the column. The countercurrent flow of dry air in the Nafion dryer was set at 2 L min^−1^. During sample collection, the nitrogen carrier gas was passed through dummy column 2. In step 2, nitrogen carrier gas was passed through the column with a flow at 50 mL min^−1^, and the column was heated to release the trapped sulfur compounds. The column temperature was held for 1 min at ambient temperature for purging, ramped up to 130°C and held for 2 min to release methanethiol, ramped up to 240°C and held for 2 min to release dimethyl sulfide, and ramped up to 270°C and held for 1 min for cleaning of the column. In step 3, nitrogen carrier gas was passed through the column with a flow at 50 mL min^−1^, and the column was cooled down to ambient temperature with a fan (Shicoh Engineering Co. Ltd., Yamato, Japan) over a period of 6 min. During step 2 and 3, sample air was passed through dummy column 1. The detection limits of the SCTS-CL were estimated as three times the baseline noise.

### 2.3. Instruments Used for Comparison

A PTR-MS (Ionicon Analytik, Innsbruck, Austria) and a GC-SCD (GC 7890 A and SCD 355, Agilent Technologies A/S, Horsholm, Denmark) were used to evaluate the results obtained by the SCTS-CL. The PTR-MS was operated under standard ion drift tube conditions applying a total voltage of 600 V and maintaining the pressure in the range of 2.1–2.2 mbar (*E*/*N* value ~135 Td). The temperature of the drift tube was controlled at 75°C, and the inlet sampling flow was adjusted to ca. 70 mL min^−1^. Instrumental background was measured on room air purified for hydrocarbon contaminants with a Supelpure HC filter (Supelco, Bellefonte, PA, USA). The sensitivity of the sulfur compounds was estimated using the rate constant for proton transfer, the estimated drift tube residence time and the mass-specific transmission factor as described by de Gouw and Warneke [21]. The detection limits of the PTR-MS were calculated as three times the standard deviation on blank samples.

The GC-SCD was equipped with a capillary column with a stationary phase of dimethylpolysiloxane (DB-1, Agilent Technologies A/S, Horsholm, Denmark). The column had a length at 60 m, an inner diameter at 0.53 mm, and a stationary phase at 5 *μ*m. The helium carrier gas flow rate was set to 10 mL min^−1^. The GC oven temperature was held for 1 min at 60°C, ramped up to 200°C at 20°C min^−1^ and held for 1 min at 200°C. The GC-SCD was equipped with a 1.0-mL sample loop. At each analysis, the sample loop was flushed with ca. 45 mL of sample air. A 5 ppm_*v*_ gas standard (Air liquide, Horsens, Denmark) containing hydrogen sulfide, methanethiol, and dimethyl sulfide was used as a one-point calibration. The detection limits of the GC-SCD were estimated as three times the baseline noise.

### 2.4. Field Samples from Pig Production

An experimental pig production facility with growing-finishing pigs (Pig Research Centre, Danish Agriculture & Food Council, Grønhøj, Denmark) was used for collection of field samples. The facility consisted of two pens with 16 growing-finishing pigs (32–107 kg) in each and was equipped with dry feeding and negative pressure ventilation with a diffuse inlet through the ceiling. The facility had one third drained floor (slatted floor with less than 10% openings) and two third slatted floor. Three air samples were collected in 10-L Tedlar bags (CEL Scientific Corporation, Santa Fe Springs, CA, USA) from the ventilation outlet of the facility. Samples were aspirated into the bags by applying negative pressure around the bags in a vacuum container. Preconditioning of the bag inner surface was done by filling the bags with sample air and emptying them twice before the final filling. All three bags were sampled within 30 min. During the sampling, the temperature in the ventilation outlet was 16.6 ± 0.2°C, and the relative humidity was 72.2 ± 0.7%. The Tedlar bags were covered with black plastic to avoid photodegradation and transported back to the laboratory for analysis. Within 3 h after collection the samples were analyzed by SCTS-CL, PTR-MS, and GC-SCD.

## 3. Results and Discussion

### 3.1. Performance of the SCTS-CL

**Figure 3:**
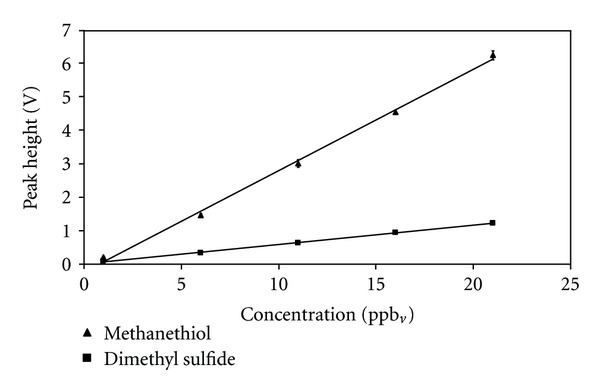
Calibration curves for single column trapping/separation and chemiluminescence detection (SCTS-CL). Methanethiol: slope = 0.3, intercept = −0.2, *R* ^2^ = 0.997; dimethyl sulfide: slope = 0.06, intercept = 0.01, *R* ^2^ = 0.999. The first calibration level is an average ± SD of seven repetitions, whereas the following four levels are average ± SD of three repetitions.

The first part of the study was concerned with the optimization and evaluation of the instrument in relation to the conditions present in sample air from pig production. An advantage of the chemiluminescence detection is the linear response [22], which makes the calibration of the instrument more simple compared to other detectors such as the pulsed flame photometric detector (PFPD) that has a quadratic response [23]. In Figure 3, calibration curves are shown for methanethiol and dimethyl sulfide in the range between 1 to 21 ppb_*v*_, which is the concentration range normally found in facilities with growing-finishing pigs [2, 3]. According to Figure 3, the response from the SCTS-CL is linear for both methanethiol (*R* ^2^ = 0.997) and dimethyl sulfide (*R* ^2^ = 0.999). Due to the high linearity of the SCTS-CL, a one-point calibration with a gas mixture containing methanethiol (measured by PTR-MS: 9.6 ppb_*v*_) and dimethyl sulfide (measured by PTR-MS: 12.6 ppb_*v*_) was used for the measurements on field samples from pig production.

### 3.2. Trapping and Desorption Property of the Silica Gel Column

The trapping efficiency of the silica gel column was estimated based on trapping of ca. 2.5 L gas mixture containing hydrogen sulfide (ca. 50 ppb_*v*_), methanethiol (ca. 10 ppb_*v*_), and dimethyl sulfide (ca. 10 ppb_*v*_). The trapping efficiency of all three compounds was above 95% in accordance with previous studies on hydrogen sulfide [13] and methanethiol [14]. Although silica gel is suitable for trapping of sulfur compounds, it requires drying of the sample air prior to collection to avoid water sorption that can have a negative influence on the recovery [13, 14]. It has previously been shown that a Nafion dryer [24, 25] and a CaCl_2_ column [26, 27] can be used to dry sample air with only a limited influence on the recovery of sulfur compounds. The Nafion dryer was chosen for the SCTS-CL because it only requires a countercurrent flow of dry air, and the changing of the drying agent during the measurements was avoided.

**Figure 4:**
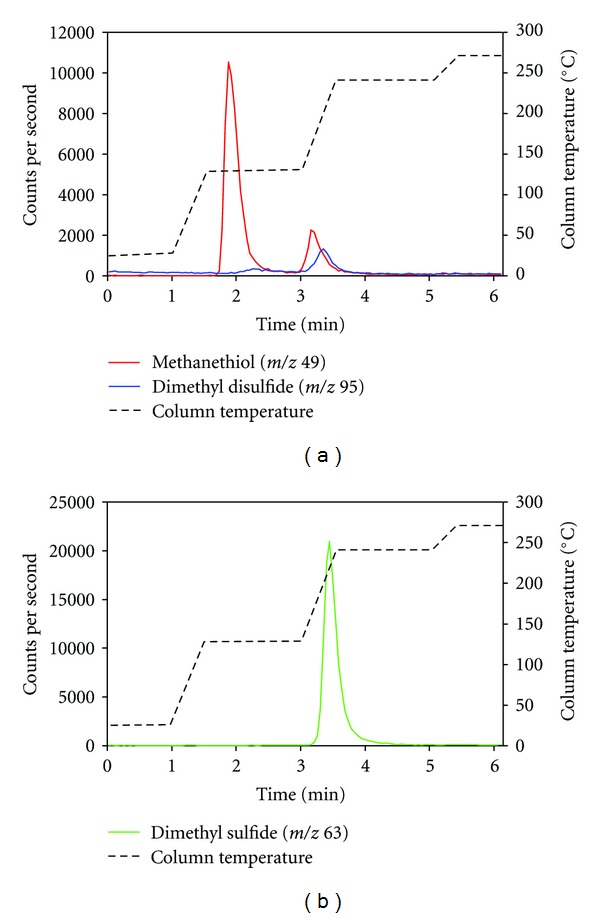
Desorption from a silica gel column loaded with ca. 600 mL of a gas mixture containing methanethiol (a) and dimethyl sulfide (b) (ca. 10 ppb_*v*_). The desorbed compounds were measured by proton-transfer-reaction mass spectrometry (PTR-MS).

Desorption of methanethiol (ca. 10 ppb_*v*_) and dimethyl sulfide (ca. 10 ppb_*v*_) measured by PTR-MS is presented in Figure 4.  Figure 4(a) shows that methanethiol was split into two peaks, whereas Figure 4(b) shows that dimethyl sulfide only resulted in one peak. The second peak of methanethiol indicates that a fraction of the molecules is trapped more firmly by the silica gel and requires a higher desorption temperature. A small peak from dimethyl disulfide is also seen in Figure 4(a), which could origin from an impurity (<0.5 ppb_*v*_) in the gas mixture or oxidation of methanethiol during the trapping and desorption process. Other studies have shown that graphitized and carbon molecular sieve sorbents and to some extent Tenax TA result in the oxidation of thiols into disulfides [11, 12]. In the study by Lestremau et al. [12], it was shown that the formation of dimethyl disulfide on a carbon molecular sieve sorbent increased from 4% of the methanethiol peak to 60% when the desorption temperature was increased from 150°C to 300°C thereby indicating that it is a thermal oxidation. In the present study, methanethiol was desorbed at 130°C and the dimethyl disulfide peak was ca. 10% of the methanethiol peak. The second peak of methanethiol and the dimethyl disulfide peak are desorbed close to the desorption temperature for dimethyl sulfide and could possibly interfere with the measurement of dimethyl sulfide.

**Figure 5:**
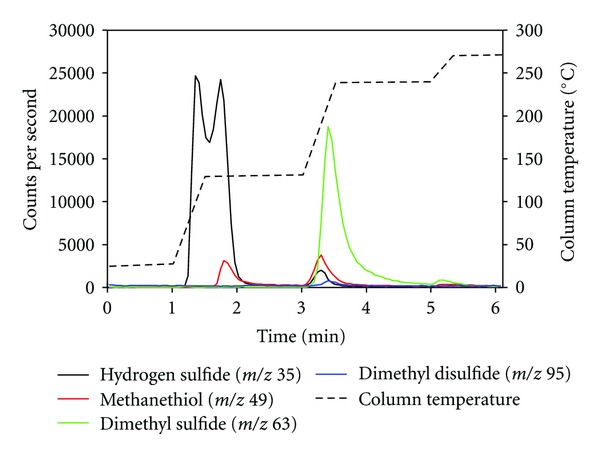
Desorption from a silica gel column loaded with ca. 600 mL of a gas mixture containing hydrogen sulfide (ca. 300  ppb_*v*_), methanethiol (ca. 10 ppb_*v*_), and dimethyl sulfide (ca. 10 ppb_*v*_). The desorbed compunds were measured by proton-transfer-reaction mass spectrometry (PTR-MS).

The high trapping efficiency of hydrogen sulfide gives a problem in relation to sample air from pig production, because the desorption temperature of hydrogen sulfide from the silica gel column is close to methanethiol [15]. The intensity of the chemiluminescent emission from the direct reaction between ozone and hydrogen sulfide is 1/100 of methanethiol [15], but at 20–30 times, higher concentrations which are normal in pig production, it will result in a detectable chemiluminescent emission that will interfere with the measurement of methanethiol. Desorption of a gas mixture with hydrogen sulfide (ca. 300 ppb_*v*_), methanethiol (ca. 10 ppb_*v*_), and dimethyl sulfide (ca. 10 ppb_*v*_) is shown in Figure 5. Figure 5 clearly shows that hydrogen sulfide is desorbed at the same time as methanethiol and that it will interfere with the measurement of methanethiol. It was also found that the first peak of methanethiol was decreased when the hydrogen sulfide concentration in the gas mixture was increased from 50 ppb_*v*_ to 300 ppb_*v*_ (results not shown), and it seems that a high hydrogen sulfide concentration also influences the trapping of methanethiol. The second peak of methanethiol was only slightly increased when the hydrogen sulfide concentration was increased from 50 ppb_*v*_ to 300 ppb_*v*_.

During measurements on the field samples, hydrogen sulfide was removed from the sample air with a small column (13 mm id × 15 cm) packed with glass wool treated with 5% Na_2_CO_3_/5% glycerin. The recovery of methanethiol (ca. 10 ppb_*v*_) and dimethyl sulfide (ca. 10 ppb_*v*_) in the column was above 95%, and the removal efficiency of hydrogen sulfide (ca. 300 ppb_*v*_) was above 99% (results not shown). The column used in the present study had a capacity for ca. ten samples, but the size of the column could be increased to handle more samples.

### 3.3. Field Samples from Pig Production

Air samples were collected from a facility with growing-finishing pigs and an intercomparison was performed among SCTS-CL, PTR-MS, and GC-SCD. To minimize the temporal variation between the three methods, sample air from the pig production was collected in Tedlar bags and transported to the laboratory for analysis within 3 h. The recovery of sulfur compounds in Tedlar bags has previously been shown to be above 90–95% several hours after collection [7–10], and the air samples should more or less reflect the concentration levels in the pig production facility. The measured concentrations of hydrogen sulfide, methanethiol, and dimethyl sulfide are presented in Table 2. The difference between SCTS-CL, PTR-MS, and GC-SCD in relation to methanethiol and dimethyl sulfide was below 10%. The relative standard deviation for measurements by SCTS-CL on field samples was 5% for methanethiol and 2% for dimethyl sulfide, which is comparable with PTR-MS (4% and 6%) and GC-SCD (7% and 1%). Although the principle of the methods is quite different, it seems that all three methods can provide reliable measurements of methanethiol and dimethyl sulfide in sample air from pig production. The measurements of hydrogen sulfide by PTR-MS and GC-SCD were also comparable, although the measurements by PTR-MS were slightly higher (ca. 7%). In Table 3, the estimated detection limits for the SCTS-CL are presented along with the PTR-MS and the GC-SCD. The SCTS-CL and the PTR-MS demonstrated significantly lower detection limits for methanethiol and dimethyl sulfide compared to the GC-SCD. The GC-SCD was based on injection of whole air samples through a sample loop and that was the main reason for the higher detection limits obtained for this method. The GC-SCD can also be combined with a pre-concentration step and in that case, it would also provide lower detection limits than shown in the present study. The detection limits for the SCTS-CL and the PTR-MS were below the estimated odor threshold values for both methanethiol and dimethyl sulfide (Table 3). Subthreshold detection limits give the opportunity to investigate the relation between odorants and the response from human noses under conditions with low concentrations such as the boundary line of an animal production facility or during the development of odor reduction technologies with a high odorant removal. Contrary to the other two methods, the PTR-MS has the advantage that it can include a wider range of odorants and provide online data with a high time resolution. The SCTS-CL is a portable instrument, but it requires 15 min for each sample which gives a relatively high temporal variation between the measurements. Furthermore, field measurements with the SCTS-CL in pig production require a large column for removal of hydrogen sulfide, which limits the usability for long-term studies. Although the SCTS-CL cannot be used as an online method such as the PTR-MS, it can still provide on-site measurements of methanethiol and dimethyl sulfide, so that storage and transportation of sample bags and adsorbent tubes or adsorptive fibers can be avoided.

It can be concluded that the SCTS-CL is a portable and low-cost alternative to the commercial methods that can be used to measure methanethiol and dimethyl sulfide in sample air from pig production, but it requires a removal of hydrogen sulfide to obtain reliable results for methanethiol. The choice of method for analysis of gaseous sulfur compounds in future studies with pig production depends on the requirements for detection limit, time resolution, and the range of odorants that can be measured.

## Figures and Tables

**Table 1:** Concentration levels (ppb_*v*_) for gas mixtures used for investigating linearity, trapping efficiency, and interferences between compounds for single column trapping/separation and chemiluminescence detection (SCTS-CL).

Gas mixture	1^a^	2^b^	3^c^	4^d^
Hydrogen sulfide	—	—	—	50–300
Methanethiol	1–21	10	—	10
Dimethyl sulfide	1–21	—	10	10

^a^Dilutions of gas standards with 100 ppm_*v*_ methanethiol (Taiyo Nippon Sanso, Tokyo, Japan) and 100 ppm_*v*_ dimethyl sulfide (Sumitomo Seika Chemicals, Osaka, Japan); ^b^Dilution of a gas standard with 5 ppm_*v*_ methanethiol (Air liquide, Horsens, Denmark); ^c^Dilution of a gas standard with 5 ppm_*v*_ dimethyl sulfide (Air liquide, Horsens, Denmark); ^d^Dilutions of gas standards with 5 ppm_*v*_ hydrogen sulfide, methanethiol, and dimethyl sulfide (Air liquide, Horsens, Denmark) and 5 ppm_*v*_ hydrogen sulfide (Air liquide, Horsens, Denmark).

**Table 2:** Measured concentrations (average ± 1 SD) of sulfur compounds in sample air from a facility with growing-finishing pigs.

Compounds	SCTS-CL^a^ ppb_*v*_	GC-SCD^b^ ppb_*v*_	PTR-MS^c^ ppb_*v*_
Hydrogen sulfide	—	310 ± 30	334 ± 33
Methanethiol	11.3 ± 0.5	11.6 ± 0.8	10.7 ± 0.5
Dimethyl sulfide	4.5 ± 0.1	5.0 ± 0.1	4.8 ± 0.1

^a^SCTS-CL: single column trapping/separation and chemiluminescence detection; ^b^GC-SCD: gas chromatography coupled with sulfur chemiluminescence detection; ^c^PTR-MS: proton-transfer-reaction mass spectrometry.

**Table 3:** Estimated detection limits and odor threshold values (OTV).

Compounds	SCTS-CL^a^ ppb_*v*_	GC-SCD^b^ ppb_*v*_	PTR-MS^c^ ppb_*v*_	OTV^d^ ppb_*v*_
Hydrogen sulfide	—	1.2	4.4	1.9
Methanethiol	0.03	1.1	0.03	0.07
Dimethyl sulfide	0.10	1.0	0.12	4.1

^a^SCTS-CL: single column trapping/separation and chemiluminescence detection; ^b^GC-SCD: gas chromatography coupled with sulfur chemiluminescence detection; ^c^PTR-MS: proton-transfer-reaction mass spectrometry; ^d^OTV: Odor threshold values were estimated as geometric mean of reported values [1].

## Abbreviations

SCTS-CL: Single column trapping/separation and chemiluminescence detection
PTR-MS: Proton-Transfer-Reaction Mass Spectrometry
GC-SCD: Gas chromatography coupled with sulfur chemiluminescence detection
ppb_*v*_: Part per billion by volume.
